# Alternative patterns of deep brain stimulation in neurologic and neuropsychiatric disorders

**DOI:** 10.3389/fninf.2023.1156818

**Published:** 2023-06-21

**Authors:** Ricardo A. Najera, Anil K. Mahavadi, Anas U. Khan, Ujwal Boddeti, Victor A. Del Bene, Harrison C. Walker, J. Nicole Bentley

**Affiliations:** ^1^Department of Neurosurgery, University of Alabama at Birmingham, Birmingham, AL, United States; ^2^Department of Neurosurgery, University of Maryland School of Medicine, Baltimore, MD, United States; ^3^Department of Neurology, University of Alabama at Birmingham, Birmingham, AL, United States

**Keywords:** theta-burst stimulation, deep brain stimulation, coordinated reset stimulation (CRS), paired pulses, closed-loop, interleaved stimulation, neuromodulation, cycling stimulation

## Abstract

Deep brain stimulation (DBS) is a widely used clinical therapy that modulates neuronal firing in subcortical structures, eliciting downstream network effects. Its effectiveness is determined by electrode geometry and location as well as adjustable stimulation parameters including pulse width, interstimulus interval, frequency, and amplitude. These parameters are often determined empirically during clinical or intraoperative programming and can be altered to an almost unlimited number of combinations. Conventional high-frequency stimulation uses a continuous high-frequency square-wave pulse (typically 130–160 Hz), but other stimulation patterns may prove efficacious, such as continuous or bursting theta-frequencies, variable frequencies, and coordinated reset stimulation. Here we summarize the current landscape and potential clinical applications for novel stimulation patterns.

## Introduction

**FIGURE 1:**
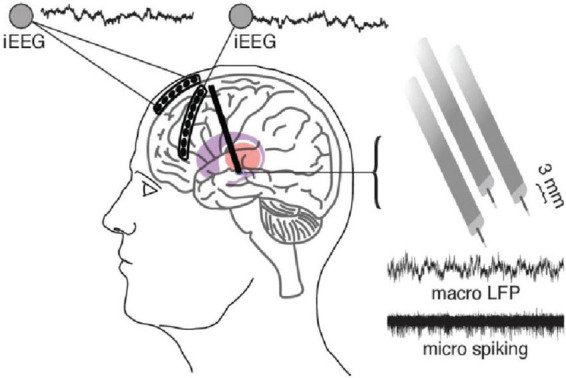
A standard DBS setup including the use of a six-contact ECoG strip over anterior PFC and eight-contact strip over lateral PFC which can be used to simultaneously record and stimulate from the DBS electrodes. DBS electrodes in this case are 3 separate macro/micro pairs which allow the recording of LFP (macro) and action potentials (micro). Adapted from Zavala et al. (2017).

**FIGURE 2:**
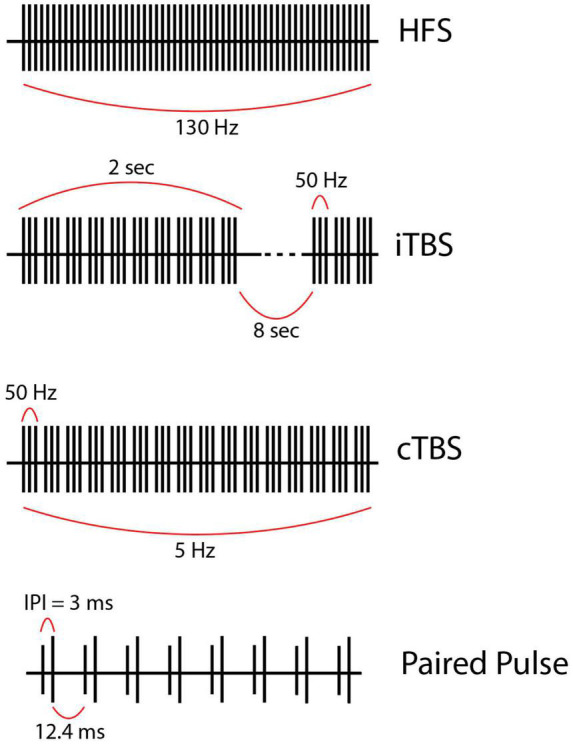
**(First row)** High frequency stimulation (HFS) is the current gold-standard and consists of continuous regular pulses with equal spacing at a high frequency (in this case 130 Hz). **(Second row)** Intermittent theta-burst stimulation (iTBS) usually given as 3 pulses at 50 Hz grouped into bursts delivered at a theta frequency (5 Hz) within a train. Each train lasts 2 s and contains 10 bursts. Trains are separated by 8 s intervals in this example but could be longer or shorter. **(Third row)** Continuous theta burst stimulation (cTBS) is like iTBS in that stimulation is delivered as three 50-Hz pulses grouped into bursts which are delivered at a theta frequency. However, the trains are not separated by a long (8 s) period. Instead, the stimulation is one long train. **(Fourth row)** Paired pulse stimulation utilizes biphasic paired pulses separated by short interstimulus intervals (ISI), the time from the first to the second stimulus within a single paired pulse (ISI = 3 ms in this example, but this value varies). Of note, the stimulation parameters used to create the figures for each alternative pattern of stimulation presented here are examples of possible parameters; however, these vary greatly across studies.

Deep brain stimulation (DBS) uses implantable depth electrodes to modulate neuronal firing in subcortical structures, eliciting downstream effects in human brain circuits (Figure 1). Intraoperative placement is followed by device programming where parameters such as pulse width, interstimulus interval (ISI), frequency, and amplitude are titrated to improve pathologic symptoms and avoid adverse side effects. Current DBS applications target motor symptoms of Parkinson’s disease (PD), essential tremor (ET), and various forms of dystonia as well as neuropsychiatric symptoms of treatment-resistant obsessive-compulsive disorder (OCD), Tourette syndrome (TS), and treatment-resistant depression (TRD) with continuous high-frequency stimulation (HFS; typically, 130–160 Hz) (Figure 2). Although its exact mechanism of action is unknown, numerous theories exist. Some studies suggest that HFS may exert its effects *via* desynchronization or reorganization of pathologic network oscillations (Wilson and Moehlis, 2015; Ozturk et al., 2021). Similarly, Rosenbaum et al. (2014) theorized that HFS works through short-term depression and decoupling of specific circuits. Other groups have theorized that HFS directly inhibits neural activity (Benazzouz and Hallett, 2000; Jensen and Durand, 2009), while some suggest the opposite, that it acts through direct excitation of neural activity (Hashimoto et al., 2003; McIntyre et al., 2004). Another theory is that HFS introduces an “information lesion,” producing similar effects to neural ablation that is also used to treat the same disease processes (e.g., PD, OCD, etc.) (Grill et al., 2004; Agnesi et al., 2013; Lowet et al., 2022), however, to date, there is no single accepted theory on the mechanism of action of HFS and further work is required to elucidate its mechanism (Hammond et al., 2008; Lozano et al., 2019).

While the benefits of HFS are well-established for motor outcomes, its impact on cognitive control is less clear. Cernera et al. (2019) reviewed studies of how DBS impacts various aspects of cognitive function and found heterogenous results within and between neurocognitive metrics based on sample size, DBS target, and disease pathology. General trends suggested declines in verbal fluency, assessed by various measures such as “phonemic fluency” (ability to recall words starting with a specific letter) and “semantic fluency” (ability to recall words related to a certain category of knowledge). These declines occurred regardless of whether the DBS target was the subthalamic nucleus (STN), globus pallidus interna (GPi), or various thalamic nuclei (Ostrem et al., 2011; Pedrosa et al., 2014; Dinkelbach et al., 2015). Other global metrics such as mini-mental state examination (MMSE) or measures of executive function such as Wisconsin Card Sorting Test (WCST) have yielded mixed results (Cernera et al., 2019). In contrast, STN low-frequency stimulation (LFS) in the theta-range has been shown to improve VF in PD patients (Lee et al., 2021). Negative impacts of HFS on verbal fluency and other aspects of cognitive function challenge the notion of expanding DBS or other neuromodulation therapies for cognitive dysfunction in patients with movement disorders and other complex neuropsychiatric diseases.

These relative shortcomings of HFS, as well as a need to avoid sensorimotor side effects, have prompted investigation of non-continuous, or “patterned,” stimulation paradigms such as theta burst stimulation (TBS) (Titiz et al., 2017; Horn et al., 2020), paired pulse stimulation (Birdno et al., 2007; Awad et al., 2021), variable frequency stimulation (VFS) (Jia et al., 2015; Zhang et al., 2019), interleaved stimulation (ILS) (Barbe et al., 2014; Kern et al., 2018), burst cycling stimulation (Velasco et al., 2007; Kuncel et al., 2012), coordinated reset stimulation (CR-DBS) (Adamchic et al., 2014), temporally optimized stimulation (Brocker et al., 2017; Okun et al., 2022), and adaptive, or “closed-loop” stimulation (aDBS/CL-DBS) (Little et al., 2016a; Piña-Fuentes et al., 2019) as alternatives. These paradigms were derived either from more physiologic patterns of neuronal firing or feedback-based systems that were computationally designed to better disrupt pathologic circuits. In this review, we provide an overview of alternative stimulation patterns and their potential applications.

## Methods

We conducted PubMed searches in November 2022 (TBS, paired pulse, VFS, CR-DBS, aDBS) and March 2023 (ILS, burst cycling, temporally optimized stimulation) to review the existing literature on alternative patterns of DBS. Search terms included: TBS {[(“Deep Brain Stimulation”[Mesh]) OR (“Deep Brain Stimulation”)] AND [(“theta burst stimulation”) OR (“intermittent theta burst”) OR (“intermittent theta burst stimulation”) OR (“continuous theta burst stimulation”) OR (“continuous theta burst”) OR (“iTBS”) OR (“cTBS”) OR (“theta burst”)]}; paired pulse {[(“Deep Brain Stimulation”[Mesh]) OR (“Deep Brain Stimulation”)] AND [(“paired pulse stimulation”) OR (“paired pulse”)]}; VFS {[(“Deep Brain Stimulation”[Mesh]) OR (“Deep Brain Stimulation”)] AND [(“variable frequency stimulation”) OR (“variable frequency”)]}; ILS {[(“Deep Brain Stimulation”[Mesh]) OR (“Deep Brain Stimulation”)] AND [(“interleaving”) OR (“interleaved stimulation”)]}; burst cycling {[(“Deep Brain Stimulation”[Mesh]) OR (“Deep Brain Stimulation”)] AND [(“cycling”) OR (“cyclical stimulation”) OR (“burst cycling”) OR (“cycling stimulation”)]}; CR-DBS {[(“Deep Brain Stimulation”[Mesh]) OR (“Deep Brain Stimulation”)] AND [(“coordinated reset stimulation”) OR (“coordinated reset”) OR (“CR-DBS”)]}; temporally optimized stimulation {[(“Deep Brain Stimulation”[Mesh]) OR (“Deep Brain Stimulation”)] AND [(“temporally optimized stimulation”) OR (“temporally optimized patterned stimulation”)]}; aDBS {[(“Deep Brain Stimulation”[Mesh]) OR (“Deep Brain Stimulation”)] AND [(“closed loop stimulation”) OR (“closed loop”) OR (“closed-loop”) OR (“adaptive stimulation”) OR (“adaptive DBS”) OR (“aDBS”) OR (“adaptive deep brain stimulation”) OR (“closed-loop stimulation”)]}.

Studies meeting these search criteria with original clinical data on human subjects undergoing DBS were included. Exclusion criteria included animal studies, computational models, reviews, non-DBS studies (i.e., transcranial magnetic stimulation, responsive neurostimulation), DBS studies using only HFS and not the alternative pattern of interest, and non-English manuscripts. Abstracts and full texts were manually screened by a single author using the inclusion and exclusion criteria mentioned above. Our goals were to qualitatively review the current literature on alternative neuromodulation techniques, and therefore we did not utilize Preferred Reporting Items for Systematic Reviews and Meta-Analyses (PRISMA) guidelines.

## Results

### Literature search

Our literature search of TBS yielded 29 studies, six of which were included (Miller et al., 2015; Titiz et al., 2017; Kim et al., 2018; Bentley et al., 2020; Horn et al., 2020; Sáenz-Farret et al., 2021). Of the excluded studies, 11 were reviews, seven were non-human animal studies, three were TMS studies without DBS, and two were editorials or commentaries with no original clinical data. The paired pulse search yielded 28 studies, of which three were included (Baker et al., 2002; Birdno et al., 2007; Awad et al., 2021). Of the excluded studies, nine were non-human animal studies, six did not use paired pulse stimulation, five were reviews, and five were TMS studies without DBS. The search for VFS resulted in 12 studies, five of which were included (Jia et al., 2015, 2017, 2018; Zhang et al., 2019; Chang et al., 2021). Of the excluded studies, two used stimulation modalities other than DBS (e.g., TMS), two were protocols for upcoming studies with no original clinical data, one was a review, one was a non-human animal study, and one did not utilize VFS. For ILS, our search yielded 27 results, of which 16 were included (Wojtecki et al., 2011; Baumann et al., 2012; Kovács et al., 2012; Barbe et al., 2014; Miocinovic et al., 2014; Ramirez-Zamora et al., 2015; Zhang et al., 2016, 2018; Kern et al., 2018; Shu et al., 2018; Aquino et al., 2019; França et al., 2019; Karl et al., 2019, 2020; Goftari et al., 2020; Zafar et al., 2021). Of the excluded studies, five were reviews, three did not use ILS, one was *in silico* (i.e., no human subjects), one was a video, and one was not in English. For burst cycling, the search found 32 studies, of which 14 were included (Montgomery, 2005; Velasco et al., 2007; Tai et al., 2011; Kuncel et al., 2012; Min et al., 2013; Boongird et al., 2016; Huang et al., 2019; Enatsu et al., 2020; Kaufmann et al., 2020; Dayal et al., 2021; Vázquez-Barrón et al., 2021; Wong et al., 2021; Dalic et al., 2022; Loeffler et al., 2022). Of the excluded studies, five did not report clinical outcomes (e.g., imaging study with fMRI), four did not use burst cycling, four did not use DBS, three were reviews, one was a conceptual study with no human subjects, and one combined VNS and DBS. For CR-DBS, our search found 32 studies, but only one was included (Adamchic et al., 2014). Of the excluded studies, 21 tested computational or theoretical models of coordinated reset, six were non-human animal studies, two were reviews, and two used stimulation modalities other than DBS (e.g., TMS). For temporally optimized stimulation, our search yielded 93 results, of which two were included (Brocker et al., 2017; Okun et al., 2022). Of those excluded, 25 were reviews, 21 did not utilize temporally optimized stimulation, 15 did not use DBS, 13 were animal studies, nine did not provide clinical outcomes, seven were computational models, and one was theoretical/conceptual with no human subjects. Finally, our literature search of aDBS yielded 576 studies, of which 31 were included (Little et al., 2013, 2016a,b; Rosa et al., 2015, 2017; Malekmohammadi et al., 2016; Cagnan et al., 2017; Herron et al., 2017a; Piña-Fuentes et al., 2017, 2019, 2020a,b; Tinkhauser et al., 2017; Arlotti et al., 2018, 2021; Swann et al., 2018; Velisar et al., 2019; Castaño-Candamil et al., 2020; Ferleger et al., 2020; He et al., 2020; Opri et al., 2020; Petrucci et al., 2020; Gilron et al., 2021; Johnson et al., 2021; Louie et al., 2021; Molina et al., 2021; Nakajima et al., 2021; Sasaki et al., 2021; Scangos et al., 2021; Cagle et al., 2022; Sarikhani et al., 2022). Of the excluded studies, 180 were reviews, 139 did not use closed-loop systems, 112 tested purely theoretical or computational models of closed-loop stimulation, 51 were non-human animal studies, seven explored stimulation modalities other than DBS (e.g., TMS), and 56 were excluded for various other reasons (e.g., editorials, commentaries, study protocols, non-English language studies). Results from the literature search are summarized in Table 1.

**TABLE 1:** Literature review of alternative patterns of stimulation.

Author (Year)	*N*	Diagnosis	Pattern of stimulation
Miller et al. (2015)	4	Epilepsy	TBS
Titiz et al. (2017)	13	Epilepsy	TBS
Kim et al. (2018)	4	Epilepsy	TBS
Horn et al. (2020)	17	PD	TBS
Bentley et al. (2020)	7	PD	TBS
Sáenz-Farret et al. (2021)	10	PD, ET, Dystonia	TBS
Baker et al. (2002)	5	PD, Epilepsy	Paired pulse
Birdno et al. (2007)	5	ET	Paired pulse
Awad et al. (2021)	17	PD, ET	Paired pulse
Adamchic et al. (2014)	6	PD	CR-DBS
Jia et al. (2015)	1	PD	VFS
Jia et al. (2017)	1	PD	VFS
Jia et al. (2018)	4	PD	VFS
Zhang et al. (2019)	1	PD	VFS
Chang et al. (2021)	1	PD	VFS
Wojtecki et al. (2011)	1	PD	ILS
Baumann et al. (2012)	1	PD, ET	ILS
Kovács et al. (2012)	4	Dystonia	ILS
Miocinovic et al. (2014)	3	PD	ILS
Barbe et al. (2014)	10	ET	ILS
Ramirez-Zamora et al. (2015)	9	PD	ILS
Zhang et al. (2016)	12	PD	ILS
Kern et al. (2018)	50	PD, ET, Dystonia	ILS
Shu et al. (2018)	1	Meige syndrome	ILS
Zhang et al. (2018)	1	Dystonia	ILS
Aquino et al. (2019)	20	PD	ILS
Karl et al. (2019)	76	PD	ILS
França et al. (2019)	17	PD	ILS
Karl et al. (2020)	20	PD	ILS
Goftari et al. (2020)	1	PD	ILS
Zafar et al. (2021)	19	PD	ILS
Montgomery (2005)	7	PD	Burst cycling
Velasco et al. (2007)	22	Epilepsy	Burst cycling
Tai et al. (2011)	1	Dystonia	Burst cycling
Kuncel et al. (2012)	10	ET	Burst cycling
Min et al. (2013)	2	Epilepsy	Burst cycling
Boongird et al. (2016)	1	Epilepsy	Burst cycling
Huang et al. (2019)	3	Chronic Pain	Burst cycling
Kaufmann et al. (2020)	23	Epilepsy	Burst cycling
Enatsu et al. (2020)	3	PD	Burst cycling
Wong et al. (2021)	10	PD	Burst cycling
Dayal et al. (2021)	6	PD, Progressive Supranuclear Palsy	Burst cycling
Vázquez-Barrón et al. (2021)	6	Epilepsy	Burst cycling
Dalic et al. (2022)	20	Epilepsy	Burst cycling
Loeffler et al. (2022)	1	Spinocerebellar Ataxia	Burst cycling
Brocker et al. (2017)	26	PD	TOPS
Okun et al. (2022)	8	PD	TOPS
Little et al. (2013)	8	PD	aDBS
Rosa et al. (2015)	1	PD	aDBS
Little et al. (2016a)	4	PD	aDBS
Malekmohammadi et al. (2016)	5	PD	aDBS
Little et al. (2016b)	10	PD	aDBS
Cagnan et al. (2017)	9	ET, Dystonia	aDBS
Tinkhauser et al. (2017)	13*	PD	aDBS
Herron et al. (2017a)	1	ET	aDBS
Rosa et al. (2017)	10	PD	aDBS
Piña-Fuentes et al. (2017)	1	PD	aDBS
Arlotti et al. (2018)	13	PD	aDBS
Swann et al. (2018)	2	PD	aDBS
Piña-Fuentes et al. (2019)	13	PD, Dystonia	aDBS
Velisar et al. (2019)	13	PD	aDBS
He et al. (2020)	3	ET	aDBS
Petrucci et al. (2020)	1	PD	aDBS
Piña-Fuentes et al. (2020a)	7	Dystonia	aDBS
Piña-Fuentes et al. (2020b)	13	PD	aDBS
Castaño-Candamil et al. (2020)	3	ET	aDBS
Opri et al. (2020)	3	ET	aDBS
Ferleger et al. (2020)	2	ET	aDBS
Sasaki et al. (2021)	12	PD	aDBS
Louie et al. (2021)	16	PD	aDBS
Molina et al. (2021)	5	PD	aDBS
Gilron et al. (2021)	5	PD	aDBS
Scangos et al. (2021)	1	TRD	aDBS
Johnson et al. (2021)	1	Dystonia	aDBS
Arlotti et al. (2021)	3	PD	aDBS
Nakajima et al. (2021)	1	PD	aDBS
Sarikhani et al. (2022)	15	ET, PD	aDBS
Cagle et al. (2022)	4	TS	aDBS

*Some patients included in other Little et al. studies.

### Theta burst stimulation

TBS began in the context of transcranial magnetic stimulation, most notably when Huang et al. applied repetitive TMS (rTMS) to modulate motor networks. TBS can be delivered in a continuous or intermittent fashion (cTBS or iTBS), consisting of repeated trains of 5 Hz bursts, each consisting of three pulses at 50 Hz. In cTBS, these bursts occur regularly at 5 Hz intervals, whereas iTBS consists of repeated trains of 5 Hz bursts for 2 s followed by an 8-s pause interval (Figure 2) (Huang et al., 2005).

Since then, TBS has been applied successfully in several neurologic disorders and has been shown to modulate neuronal activity and associated cognitive functions. Our group found that subcortical iTBS can evoke theta oscillatory activity, known to be important in cognitive domains such as decision making and memory (Zavala et al., 2017; Jones et al., 2020; Vivekananda et al., 2021; Zeng et al., 2021; Chen et al., 2022), in connected dorsolateral prefrontal cortex (Bentley et al., 2020). Others expanded on this and found that cortical TBS evokes frequency-specific oscillations (Solomon et al., 2021), such that 5 Hz TBS maximally increases 5 Hz power. Behavioral modulation with TBS is evident with studies showing improvements in visual-spatial memory with fornix stimulation (Miller et al., 2015) and declarative memory with entorhinal stimulation (Titiz et al., 2017). Titiz et al. additionally states that delivering stimulation through microwires as opposed to large DBS stimulation electrodes can yield improved outcomes in various cognitive functions such as face categorization and learning rate during reinforcement learning. Kim et al. (2018) found differential effects on which components of memory were recalled by identifying and stimulating network nodes that were involved in these processes using theta coherence as a marker. For example, they were able to specifically impair ability to recall spatial details of a memory while sparing recall of temporal details.

### Paired-pulse stimulation

Paired stimulus pulses have been used for decades to investigate neural refractoriness, augmentation, and plasticity (Zucker and Regehr, 2002; Bueno-Junior and Leite, 2018). These paradigms typically consist of a “conditioning” pulse followed by a “test” pulse separated by a specific ISI (Figure 2). The goal is to record short-term changes in neural activities that propagate through the engaged network. At certain ISIs, paired pulses likely increase presynaptic influx of calcium ions (Ca^2+^), and, in turn, enhance secretory exocytosis of neurotransmitters into the synaptic cleft (Zucker and Regehr, 2002). Paired pulses can be studied rapidly and elicit diverse neural responses, such that they are a versatile tool to study network dynamics and mechanisms of action in various circuits of interest (Paek et al., 2013), including the STN, GPi (Baker et al., 2002; Yamawaki et al., 2012; Awad et al., 2020; Campbell et al., 2022), and the ventrolateral thalamus (Anderson et al., 2006; Birdno et al., 2007).

Baker et al. (2002) demonstrated that paired-pulse stimulation is feasible in humans in both PD and drug-resistant epilepsy (*n* = 4), using externalized DBS leads and custom external pulse generators. Awad et al. (2021) investigated the mechanism of action of DBS using paired pulses in PD (*n* = 8) and essential tremor (ET; *n* = 6). The authors validated the neural origin of short- and long-latency tissue responses and suggested that paired DBS pulses increase local tissue electrophysiologic synchrony. Specifically, they observed that certain properties (e.g., long latency, amplitude) of later oscillatory response [i.e., evoked resonant neural activity (ERNA)] mirrored properties consistent with orthodromic synaptic activity and vesicle release. Moreover, ERNA was faster at specific ISIs (∼5–10 ms), which corresponded with the timing of therapeutic stimulation frequencies (100–200 Hz), suggesting that the timing of prior effective stimulation may facilitate recruitment of subsequent responses within the same local circuit. Thus, the authors concluded that ERNA evokes short-term facilitation/plasticity in the STN-GPi circuit in various movement disorders and showed a positive correlation with both clinical efficacy and resting beta power. Campbell et al. (2022) investigated the effects of pulse timing on DBS evoked potentials within the basal ganglia-thalamocortical (BGTC) circuit using a wide range of ISIs in a paired pulse stimulation paradigm in patients with PD (*n* = 5). They demonstrated that ISIs with frequencies > 250 Hz significantly impacted evoked potentials recorded from the STN (*via* DBS leads) and motor cortex (*via* scalp EEG). Specifically, ISIs from 1.0 to 3.0 ms produced enhanced activation (i.e., greater wavelet amplitude), while ISIs outside of this range yielded no significant changes.

Birdno et al. (2007) evaluated the effects of pulse-to-pulse changes in DBS frequency in ET (*n* = 5) using biphasic paired pulses (ISI 0.3–7.7 ms). They applied monopolar stimulation for 40–60 s in blinded subjects and found that tremor suppression decreased as “IPI_diff_” increased. In other words, as the ISI increased and/or the time between each pair of pulses increased, paired pulse stimulation became less effective than continuous HFS for treatment of ET [for more on “IPI_*diff*_,” see Birdno et al. (2007)]. Furthermore, continuous HFS at 130 Hz with regular temporal spacing was more effective at reducing tremor than paired pulse stimulation with irregular ISIs at the same overall rate (130 pulses per second). This and similar studies suggest that DBS is dependent not only on the average frequency but also on the temporal spacing of DBS pulses (Birdno et al., 2008).

### Variable frequency stimulation

While HFS DBS effectively treats PD motor symptoms (i.e., bradykinesia, rigidity, and tremor) (Perestelo-Pérez et al., 2014; Xie et al., 2016), it is less beneficial for levodopa-unresponsive elements of gait dysfunction, freezing of gait (FOG), postural instability, and speech disorders (e.g., dysarthria, hypophonia) (Benabid et al., 2009; Schlenstedt et al., 2017). Moreover, HFS may exacerbate existing symptoms or cause side effects such as decreased verbal fluency (Parsons et al., 2006). Studies investigating LFS (<100 Hz) provide evidence for greater improvements in speech, dysphagia, gait dysfunction, and FOG versus HFS (Yu et al., 2020; Conway et al., 2021; Razmkon et al., 2022). Additionally, a double-blind study found that LFS was associated with improved speech intelligibility, prosody, and both semantic and phonemic verbal fluency (Grover et al., 2019; Lee et al., 2021). One meta-analysis reported that while HFS did have more pronounced effects on tremor reduction, LFS was significantly more effective in treatment of gait dysfunction, FOG, and akinesia (Su et al., 2018). However, some studies show that the benefits of LFS versus HFS decrease with long-term use and may depend on PD phenotype (Vallabhajosula et al., 2015; Xie et al., 2018).

**FIGURE 3:**
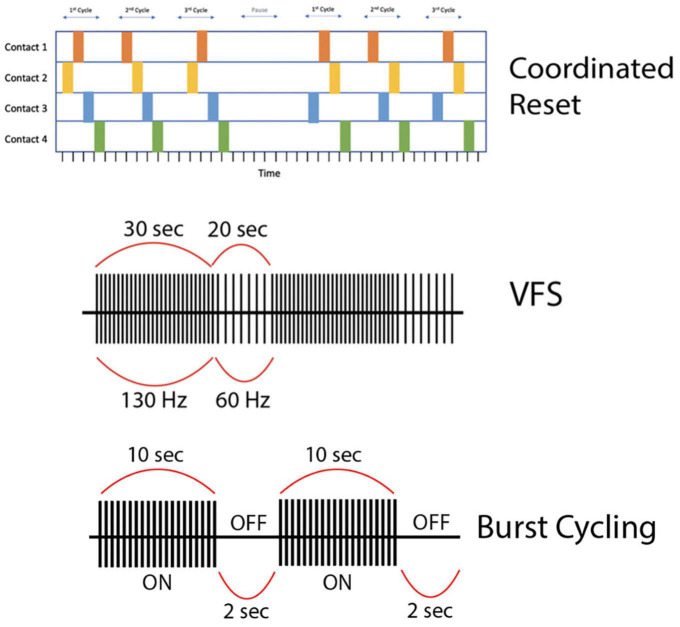
**(First row)** This is a representation of high-frequency (HF) Coordinated-Reset (CR) Deep Brain Stimulation (DBS). Specifically, we depict a shuffling pattern of stimulation, during which each cycle of stimulation has a new order in which the electrode contacts are stimulated. We also show how the CR-DBS paradigm typically consists of a series of ON cycles interspersed with OFF cycles. **(Second row)** This is a representation of variable frequency stimulation (VFS). During a stimulation cycle lasting 50 s, stimulation alternates between high frequency (130 Hz) and low frequency (60 Hz). **(Third row)** This is a representation of burst cycling stimulation with alternating ON/OFF periods (10 s/2 s). Of note, the stimulation parameters used to create the figures for each alternative pattern of stimulation presented here are examples of possible parameters; however, these vary greatly across studies.

To address these issues, researchers have experimented with VFS, with the rationale that combined elements of HFS and LFS might more optimally improve both appendicular and axial symptoms of PD. The VFS paradigm alternates between high (>100 Hz) and low (<100 Hz) frequencies within a stimulation cycle (<60 s) (Figure 3). Three studies from a single group (*n* = 6) demonstrated the feasibility of VFS in the context of bilateral STN DBS for PD. They delivered VFS, cycling between HFS and LFS patterns with a variable duty cycle (≤300 s; PINS Medical, Beijing), in patients who had previously undergone HFS DBS optimization without resolution of axial motor symptoms or with development of side effects. Their VFS paradigm involved 10–50 s trains of alternating HFS and LFS (e.g., 130 Hz for 30 s and 60 Hz for 20 s per 50 s cycle). VFS increased gait speed, mitigated FOG, and improved bradykinesia, while tremor and rigidity either remained stable or further improved compared to HFS alone (Jia et al., 2015, 2017, 2018). In two cases, HFS-associated decline in verbal fluency and dysarthria resolved with VFS without diminishing HFS-related improvement in primary appendicular motor symptoms (Jia et al., 2017; Zhang et al., 2019). An additional case report showed improvement in freezing and limb dyskinesia in a single patient with bilateral STN and GPi leads programmed for VFS, which did not occur with HFS alone (Chang et al., 2021). In ET, HFS was superior to LFS for tremor reduction but worsened verbal fluency, while LFS was more effective at enhancing verbal fluency compared to DBS-OFF and HFS (*p* = 0.0119), but had no significant incremental effects on tremor (Pedrosa et al., 2014) and in some cases actually exacerbated the tremor (Pedrosa et al., 2013). Thus, while VFS appears promising in PD, its potential seems less promising for ET with the frequency range and duty cycles previously applied in PD patients. Larger, prospective randomized controlled trials of VFS versus HFS for PD are currently underway (Jia et al., 2019; Karl et al., 2019).

### Interleaved stimulation

Standard DBS contact configurations are either monopolar, where the contact serves as the cathode, or bipolar, where two different contacts serve as an anode and cathode, respectively. The shape or coverage of the generated stimulation field can be modified by alternating between these two settings. Suboptimal position of an electrode can result in inadequate coverage of the target with overlap into anatomic regions that cause side effects. If adjusting between monopolar, double monopolar, bipolar, and double bipolar stimulation or changing stimulation parameters fails to achieve therapeutic effect, ILS can be used to contour the stimulation field and avoid unwanted overlap with non-target regions. ILS is a novel strategy that is supported in newer- generation DBS electrodes and involves rapidly alternating between two different stimulation settings using two different contacts on the same lead. Contacts can be programmed to deliver different amplitudes and pulse widths, however, the combined frequency is set to a maximum of 250 Hz per device by manufacturers to avoid potentially harmful charge delivery. ILS has been successfully used in PD to improve rigidity, bradykinesia, tremor (Wojtecki et al., 2011; Miocinovic et al., 2014; Ramirez-Zamora et al., 2015; Zhang et al., 2016; França et al., 2019; Karl et al., 2019), and gait (e.g., FOG) symptoms (Zhang et al., 2016; Karl et al., 2019; Zafar et al., 2021), as well as to decrease unwanted side effects such as dysarthria (Wojtecki et al., 2011; Ramirez-Zamora et al., 2015; Zhang et al., 2016), dyskinesias (Ramirez-Zamora et al., 2015; Zhang et al., 2016; Kern et al., 2018; Aquino et al., 2019; França et al., 2019; Goftari et al., 2020), and diplopia or paresthesias (Miocinovic et al., 2014).

Baumann et al. described a case of concomitant PD and ET where initially one set of stimulation parameters relieved PD but not ET symptoms and another set of parameters did the opposite. However, ILS using alternating unipolar pulses with different amplitudes at opposite poles of the STN and ventrolateral anterior thalamic region relieved both PD and ET symptoms (Baumann et al., 2012). Barbe et al. reported the successful use of ILS in several ET patients to relieve stimulation-induced dysarthria while maintaining therapeutic tremor suppression (Barbe et al., 2014).

Kovács et al. (2012) published the first case series (*n* = 4) of successful ILS use to treat dystonia in patients who had not previously responded to conventional high-frequency pallidal DBS. Zhang et al. (2018) later published a case report with similar findings, supporting the need for further exploration of ILS use in dystonia patients not responding to HFS DBS. Finally, Shu et al. (2018) described a case of pallidal DBS for Meige syndrome where ILS settings greatly improved the patient’s symptoms compared to HFS DBS.

### Burst cycling

Unlike traditional continuous HFS DBS (Deuschl et al., 2006; Okun et al., 2009), a temporal pattern of “burst cycling” stimulation ON and OFF has also gained traction (Figure 3). This pattern has been tested in a variety of neurologic disorders with varying results. For PD-associated tremor, there is some evidence that cycling stimulation (10s/1s or 30s/5s ON/OFF) in the STN or thalamus may help prevent tremor rebound and tolerance to DBS (Enatsu et al., 2020). A later study found that the implementation of a cycling stimulation pattern improved FOG in one PD patient who had developed tolerance to DBS (Dayal et al., 2021). On the other hand, Wong et al. (2021) did not find significant differences between cycling and conventional HFS in the treatment of FOG in patients with PD. Montgomery et al. found that continuous HFS of the STN provided greater symptom relief than burst cycling, showing a linear relationship between cycling interval and motor performance, with increasing efficacy of cycling from 0.1 to 0.5 s, both of which were inferior to continuous HFS. However, their results were likely underpowered and did not provide definitive conclusion (Montgomery, 2005). While stimulating the thalamus in the treatment of postural tremor, Kuncel et al. (2012) similarly found that cycling parameters more closely matching continuous HFS produced the greatest reduction in tremor power.

In dystonia, burst cycling has shown some therapeutic effects while increasing battery life and reducing the frequency of battery replacements (Tai et al., 2011). Loeffler et al. employed a unique cycling stimulation paradigm in an attempt to treat tremor in a patient with FGF-14 associated spinocerebellar ataxia. Their cycling paradigm used HFS (180 Hz) during the day and switched to LFS at night (30 Hz). This group found that alternating high and low frequency in the daytime and nighttime, respectively, led to a significantly better tremor response than with stimulation OFF at night (Loeffler et al., 2022). Burst cycling has also been used to treat non-motor conditions, the most common being various forms of drug-related epilepsy (DRE). Velasco et al. (2007) applied a burst cycling paradigm consisting of 1 min ON and 4 min OFF in the centromedian nucleus of the thalamus to effectively treat generalized tonic-clonic seizures and atypical absence seizures in Lennox-Gastaut syndrome. A double-blind, randomized control trial (ESTEL trial) found cycling 145 Hz for 1 min ON and 5 min OFF significantly reduced seizures in Lennox-Gastaut syndrome compared to no stimulation (Dalic et al., 2022). The stimulation parameters selected for this trial were in large part influenced by a prior double-blind, randomized control trial (SANTE trial) that found a significant reduction in seizure frequency in patients with refractory epilepsy using cycling stimulation of the thalamus (Fisher et al., 2010). These findings have been corroborated by other groups, such as Boongird et al. (2016), who showed a 60% seizure reduction 24 months after surgery with a 145 Hz 1 min ON, 5 min OFF cycling paradigm. A similar cycling paradigm (1 min ON, 4 min OFF) was used with success to stimulate the subiculum in six patients with mesial temporal lobe epilepsy associated with hippocampal sclerosis (Vázquez-Barrón et al., 2021). This is in line with conclusion of another study with mesial temporal lobe epilepsy patients where amygdalohippocampal cycling stimulation (1 min ON, 3 min OFF) resulted in seizure reductions at 2 and 18 months (Min et al., 2013). When directly comparing anterior nucleus of thalamus cycling stimulation to conventional stimulation in DRE patients, Kaufmann et al. (2020) observed no significant difference in seizure frequency; however, they found an increase in restlessness with increased cycling frequency (shorter duration OFF period). Finally, cycling stimulation has been shown to be useful to avoid after-discharges and resultant seizures when using anterior cingulate DBS to treat chronic pain (Huang et al., 2019).

### Coordinated reset

Synchronous neuronal firing is thought to underlie critical behavioral processes such as memory formation. In fact, recent studies suggest that greater neuronal synchrony between mesial temporal lobe structures correlates with improved memory performance (Jutras and Buffalo, 2010). However, hypersynchronous activity in neural circuits may also underlie pathological brain states, such as PD, epilepsy, and tinnitus (Ebert et al., 2014). For example, synchronization among populations of neurons in the thalamus and basal ganglia in PD and ET is associated with characteristic pathological movements that are typically seen in these movement disorders. More importantly, studies exploring pharmacological interventions with dopaminergic drugs (e.g., Levodopa) and/or surgical interventions with DBS have recently shown a direct correlation between reduced synchronized oscillatory activity in the β-band (8–35 Hz) and improved motor performance, suggesting the potentially critical role that synchronized neuronal activity may play in the pathogenesis of movement disorders such as PD (Chung et al., 2018).

CR-DBS is an alternative stimulation protocol that delivers intermittent high-frequency bursts to disrupt hypersynchronous neuronal firing (Figure 3). Typical CR-DBS parameters consist of high-frequency trains with an intra-burst frequency of 130 Hz (current: 2.0–4.0 mA), cycle repetition rate of 3–20 Hz, and pulse width ranging from 60 to 120 μs. Each pulse train typically consists of three to five pulses and the total duration of a pulse train ranges from 23 to 38 ms (Adamchic et al., 2014). This pattern of stimulation can be applied to electrode contacts in either a serial (non-shuffled) or shuffled (random order stimulation of electrode contacts) fashion (Wang et al., 2022). Developed in 2003, the rationale of CR-DBS is to desynchronize neuronal populations in the basal ganglia-motor cortical circuit to achieve therapeutic benefits in movement disorders such as PD (Tass, 2003). In a proof-of-concept study by Adamchic et al. (2014), six PD patients underwent bilateral implantation of quadripolar DBS electrodes in the STN. The patients were evaluated before and after once-daily 2-h CR-DBS treatments for a total of 3 days. Their CR-DBS stimulation protocol (detailed above) was specifically delivered to the three distal contacts of the electrode. They demonstrated that after 3 days of CR-DBS treatment, there was a significant reduction in β-band activity, with a mean reduction in β-power of 42% (*p* = 0.03), and an associated significant improvement in motor function [mean Unified Parkinson’s Disease Rating Scale (UPDRS) score reduction of 58%, *p* = 0.03] (Adamchic et al., 2014). It is important to note that this study was limited by a lack of comparison to traditional HFS DBS, however, it is the first to show the therapeutic efficacy of CR-DBS in managing PD motor symptoms.

### Temporally optimized stimulation

Traditional HFS DBS parameters are selected empirically based on individual patient testing and programmer experience. Newer alternative patterns have focused on using biomarkers or computational models to predict the “best settings” for a stimulation paradigm. Temporally optimized stimulation is a non-standardized computationally optimized pattern of DBS. In theory, a temporally optimized pattern of stimulation would significantly reduce energy consumption and frequency of implantable pulse generator (IPG) replacement, improving overall clinical outcomes.

In 2013, Brocker et al. (2013) tested a wide variety of temporally irregular DBS patterns in PD patients (*n* = 10) while assessing motor outcomes *via* a finger-tapping task in the operating room. This group found that stimulation pattern, and not rate, more significantly impacted DBS efficacy. Interestingly, they employed several patterns that relieved motor symptoms more effectively than temporally regular HFS, including “absence,” “presence,” “unipeak,” and “uniform,” with varying pulse entropy and frequencies (see Brocker et al., 2013 for detailed review).

Later, in a proof-of-concept study, Brocker et al. used model-based computational evolution to develop a temporally optimized stimulation paradigm (Brocker et al., 2017). They coupled a model of the basal ganglia with a genetic algorithm (GA) thought to operate similarly to evolution, with “natural selection” occurring to optimize stimulation. Three patterns were tested: temporally regular 185 Hz HFS, temporally regular 45 Hz LFS, and the optimized, GA pattern of stimulation, with an average frequency of 45 Hz. In a finger-tapping task in bradykinesia-dominant PD patients (*n* = 4), there was no significant difference in the rate and regularity of finger tapping between the HFS and GA groups, though both improved these parameters compared to baseline. Similarly, in the tremor-dominant PD subjects (*n* = 4), they found no significant difference in the reduction of tremor between the HFS and GA groups, though both significantly decreased tremor compared to baseline. Thus, despite significant study limitations, GA was tentatively deemed equivalent to HFS in terms of efficacy, but superior to HFS in terms of energy efficiency and battery life preservation (Brocker et al., 2017).

Recently, in a prospective, randomized, cross-over, multi-center feasibility study (*n* = 26), Okun et al. tested both versions of temporally optimized stimulation (TOPS) from the two aforementioned Brocker et al. studies [TOPS1 (Brocker et al., 2017) and TOPS2 (Brocker et al., 2013)] (Okun et al., 2022). They defined TOPS as pulse trains with a repeating sequence of non-regular and non-random intervals between pulses. TOPS2 used a long burst sequence (inter-burst interval ∼50 ms) followed by a short burst sequence (inter-burst interval ∼5 ms), with an average frequency of ∼158 Hz [see “absence” in Brocker et al. (2013)]. Like Brocker et al., this group found that TOPS reduced motor symptoms as effectively as conventional HFS DBS (Okun et al., 2022). As this was a safety and feasibility study, the results were not powered to provide statistically significant conclusion on efficacy. Nonetheless, the promising findings of potential non-inferiority of TOPS vs. HFS, with the added knowledge that TOPS is more energy efficient, should lead to well-powered randomized, controlled clinical trials comparing TOPS against HFS.

### Closed-loop and adaptive stimulation

Current DBS systems use open-loop stimulation in which stimulation is always ON. Closed-loop adaptive neuromodulation relies on a control signal to initiate changes in stimulation parameters in real-time. Biomarker-controlled DBS may be useful, as symptoms fluctuate throughout the day in many neurologic and neuropsychiatric disorders. In PD, tremors occur at rest and rigidity occurs with the onset of movement (Reich and Savitt, 2019), whereas in ET, tremor occurs with movement (Shanker, 2019). In neuropsychiatric conditions such as treatment-resistant OCD, symptoms fluctuate throughout the day, with obsessive fixation alternating with ritualistic compulsive behaviors, all of which vary widely from patient to patient (Goodman et al., 2014). In treatment-resistant depression (TRD), symptoms may be more pronounced in times of stress and minimally present at rest, though this disease is also characterized by highly heterogeneous symptoms (Filatova et al., 2021). In Tourette syndrome (TS), tics emerge frequently when the patient is under high stress, but manifest in different areas of the body, with variable duration and intensity (Jankovic and Kurlan, 2011). Thus, neuromodulation strategies for these diseases might be more effective, more efficient, or better tolerated with on-demand stimulation paradigms. The goal of adaptive DBS (aDBS) is to treat each patient’s constellation of symptoms in an individualized manner, to reduce stimulation-induced side effects (including during sleep), and to prolong battery life through more efficient energy consumption.

The majority of studies in aDBS focus on PD (Little et al., 2013, 2016a,b; Rosa et al., 2015, 2017; Malekmohammadi et al., 2016; Piña-Fuentes et al., 2017, 2019, 2020b; Tinkhauser et al., 2017; Arlotti et al., 2018, 2021; Swann et al., 2018; Velisar et al., 2019; Petrucci et al., 2020; Gilron et al., 2021; Molina et al., 2021; Nakajima et al., 2021; Sasaki et al., 2021; Sarikhani et al., 2022). Several groups suggest that STN aDBS is equivalent or non-inferior to open-loop or constant DBS (cDBS) in reducing the UPDRS score in PD (Little et al., 2016a; Piña-Fuentes et al., 2017, 2020b; Rosa et al., 2017; Arlotti et al., 2018; Swann et al., 2018; Velisar et al., 2019; Sasaki et al., 2021; Sarikhani et al., 2022). Some studies found greater score reductions with aDBS compared to cDBS (Little et al., 2013, 2016b; Rosa et al., 2015; Malekmohammadi et al., 2016). A potentially important limitation of these studies is that they were conducted with externalized leads and experimental pulse generators in a controlled research environment in the immediate post-lead-implantation period. Further studies are needed to demonstrate the generalizability of these findings in long-term studies and non-clinical environments.

Adaptive DBS is also of interest in neurocognitive aspects of PD. Little et al. (2016b) investigated a binary (ON-OFF) aDBS paradigm in eight PD patients with bilateral STN leads. They developed a threshold-based algorithm using beta oscillations (13–30 Hz) that switched ON and OFF automatically with a ramp up/down time of 250 ms (Little et al., 2013). They administered a speech intelligibility test (SIT) at baseline, during cDBS, and during aDBS (15-min duration) to measure speech-related side effects and improvement. They found that aDBS was associated with improved SIT scores compared to both baseline and cDBS (baseline SIT 67.9%; aDBS 70.4%; cDBS 60.5%; *p* = 0.02).

In ET, several studies report outcomes of unilateral VIM or zona incerta aDBS combined with recordings from subdural strip electrodes over primary motor cortex (Herron et al., 2017a,b; Castaño-Candamil et al., 2020; Ferleger et al., 2020; He et al., 2020, 2021; Opri et al., 2020). Two studies showed greater tremor suppression with aDBS (Castaño-Candamil et al., 2020; Ferleger et al., 2020) and one study showed aDBS to be equivalent or non-inferior to cDBS (Opri et al., 2020). On the other hand, Herron et al. (2017a) demonstrated decreased tremor control with aDBS compared to cDBS.

Dystonia presents both opportunities and challenges for aDBS applications. Piña-Fuentes et al. (2020a) found that short-term GPi aDBS (*n* = 7) did not lead to acute changes in low frequency oscillations (4–12 Hz) or to any significant clinical changes. However, dystonia typically has a delayed response to cDBS such that aDBS effects could be difficult to extrapolate in the context of short-term stimulation. DBS programming in dystonia patients is often more complex than for PD or ET, such that robust, effective closed loop stimulation strategies could play a useful role in these patients.

In neuropsychiatric disease, aDBS has been described in TRD (Scangos et al., 2021) and TS (Cagle et al., 2022). In TRD, Scangos et al. (2021) demonstrated safety and feasibility of a fully integrated aDBS system in a single patient. They found that bilateral amygdala gamma power is correlated to elevated symptom severity with high reproducibility. Stimulating at the VC/VS, they demonstrated improved depressive symptoms correlating to reduced amygdala gamma power in two of five stimulation trials. Cagle et al. (2022) compared aDBS to cDBS in four TS patients with bilateral centromedian-parafascicular complex thalamic leads. Using a subdural strip overlying M1 and thalamic leads, they found increased low-frequency thalamic power (3–10 Hz) at the onset of involuntary tics that was not present during voluntary movements. Though there was no statistically significant difference between the two, both cDBS and aDBS significantly reduced symptoms compared to DBS OFF, suggesting possible non-inferiority of aDBS versus cDBS in TS. As in all indications, seeking a suitable control signal presents challenges, but recent studies show that aDBS for OCD may be on the horizon (Provenza et al., 2019, 2021). For example, Provenza et al. (2021) recently found that delta-band (0–4 Hz) power showed a strong negative correlation with symptom severity in five patients with OCD implanted with sensing-capable IPGs and bilateral ventral capsule/ventral striatum (VC/VS) electrodes. Though this remains to be tested with aDBS, this finding may represent a suitable biomarker. While aDBS is of increasing interest, these studies are all limited by small sample sizes and require more robust investigation.

## Discussion

As our familiarity with continuous high-frequency DBS, the gold-standard stimulation paradigm in the treatment of both neurologic and neuropsychiatric disorders, grows, clinicians and scientists increasingly recognize the need for programming optimization and improved efficacy, which has led to the development of numerous alternative patterns of stimulation. In this review, we provide a snapshot of current human evidence supporting these patterns–including TBS, paired pulse, VFS, ILS, burst cycling, CR-DBS, TOPS, and aDBS–as possible alternatives to HFS DBS. With small case series in most cases, the existing literature is not sufficient to reach definitive conclusion regarding non-inferiority/superiority of an alternative pattern versus HFS DBS. However, based on our findings, in addition to these being safe and technically feasible, they generally present two specific advantages over HFS DBS: (1) optimization leads to a more energy-efficient delivery of stimulation, prolonging battery life and reducing the frequency of costly IPG replacements; (2) non-continuous paradigms have the potential to simultaneously deliver therapeutic levels of stimulation, treating both motor and non-motor symptoms of various neurologic and neuropsychiatric diseases, while mitigating stimulation-induced side effects. By the temporal nature of their designs (i.e., non-continuous), many of the alternative patterns of stimulation discussed here would draw less power from an IPG than continuous HFS DBS when delivering stimulation. In TBS, specifically iTBS, stimulation trains or bursts are followed by pauses (DBS OFF periods) (Huang et al., 2005). Similar to iTBS, VFS presumably would draw less electrical energy than HFS alone because of the alternating periods of LFS and HFS in a given cycle. However, though theoretically iTBS and VFS would consume less battery life than cTBS or HFS DBS alone, direct evidence of this is limited. Continuous TBS appears to improve motor PD symptoms, although the therapeutic threshold is higher with low intraburst frequencies (∼50 Hz vs. 100 Hz) (Horn et al., 2020). The impact of burst cycling on battery longevity has been directly investigated, and one of the main motivations for using burst cycling stimulation over conventional continuous HFS has been to conserve device battery and decrease the frequency of IPG replacements (Kuncel et al., 2012; Wong et al., 2021). In the case of TOPS and aDBS battery life is conserved in a more indirect approach, using computational modeling or biomarker-driven auto-regulating systems, respectively, which ultimately leads to more efficient energy consumption. Studies of ILS have shown conflicting results, with some indicating that ILS may lead to higher energy consumption and decreased battery life (Kern et al., 2018; Karl et al., 2019), and others showing evidence that certain ILS settings may conserve energy relative to continuous HFS DBS (Karl et al., 2020). In contrast to the primary motivation of increasing battery longevity, several studies focused on the potential mitigation of stimulation-induced side effects provided by these non-traditional paradigms, specifically VFS, ILS, burst cycling, CR-DBS, and aDBS. Many VFS studies showed improvement in gait dysfunction, FOG, and stimulation-induced speech disorders (e.g., dysarthria, hypophonia) with the use of VFS. Additionally, ILS studies observed improvements in rigidity, bradykinesia, tremor, and gait (e.g., FOG) symptoms as well as mitigation of unwanted side effects such as dysarthria, dyskinesias, diplopia, and paresthesias.

Finally, we found that some alternative patterns of stimulation may mimic a more physiologic neuronal firing pattern, which may be an explanation for why some alternative stimulation patterns seem to better address cognitive or memory-related symptoms of movement disorders and other neuropsychiatric disorders. For example, there is evidence that dorsolateral prefrontal cortex TBS can modulate networks involved in mood and cognitive function (e.g., memory). The ability to increase theta-power on-demand may provide a way to modulate specific neuronal populations, since it is believed that higher power theta oscillations can entrain high-frequency activity more efficiently (Kahana et al., 1999). Indeed, theta-gamma coupling (and theta band activity entraining single neuron firing) has functional relevance for cognition in humans across multiple domains including memory and decision making.

We acknowledge that our broad review of alternative patterns of stimulation has some methodological limitations. The most important limitation of this study is that, though we hope to review all alternative patterns of stimulation with published human data in a comprehensive manner, we are unable to account for those patterns for which we did not search. Additionally, there are likely some alternative patterns which may not have a standard naming convention and thus are difficult to review. For example, Akbar et al. (2016) conducted a study using square biphasic pulses and other irregular pulse patterns in PD (*n* = 8) and ET (*n* = 3) patients. Briefly, their findings showed that certain non-conventional patterns of stimulation may extend battery life and minimize stimulation-associated side effects. The goal of their randomized, blinded pilot study was to provide a framework for rigorously testing non-HFS patterns of stimulation; however, they specify that further testing is needed to assess efficacy of alternative patterns of stimulation and compare them to conventional HFS (Akbar et al., 2016).

## Conclusion

Alternative patterns of stimulation such as theta burst, paired pulse, variable frequency, interleaving, burst cycling, coordinated reset, temporally optimized stimulation, and adaptive DBS are promising novel patterns of stimulation that may provide improved efficacy for both motor and non-motor symptoms of neurologic and neuropsychiatric disorders. In doing so, these patterns may also extend battery life and lead to fewer replacements, ultimately improving quality of life for these patients. However, current evidence is limited and warrants rigorous trials before implementing in the clinical domain.

## Author contributions

RN, AM, AK, UB, VD, HW, and JB: conceptualization, writing—original draft preparation, and writing—review and editing. VD, HW, and JB: supervision. All authors have read and agreed to the published version of the manuscript.
